# Mapping the Economic Costs and Benefits of Conservation

**DOI:** 10.1371/journal.pbio.0040360

**Published:** 2006-10-31

**Authors:** Robin Naidoo, Taylor H Ricketts

**Affiliations:** Conservation Science Program, World Wildlife Fund, Washington, District of Columbia, United States of America; The David and Lucile Packard Foundation, United States of America

## Abstract

Resources for biodiversity conservation are severely limited, requiring strategic investment. Understanding both the economic benefits and costs of conserving ecosystems will help to allocate scarce dollars most efficiently. However, although cost-benefit analyses are common in many areas of policy, they are not typically used in conservation planning. We conducted a spatial evaluation of the costs and benefits of conservation for a landscape in the Atlantic forests of Paraguay. We considered five ecosystem services (i.e., sustainable bushmeat harvest, sustainable timber harvest, bioprospecting for pharmaceutical products, existence value, and carbon storage in aboveground biomass) and compared them to estimates of the opportunity costs of conservation. We found a high degree of spatial variability in both costs and benefits over this relatively small (~3,000 km^2^) landscape. Benefits exceeded costs in some areas, with carbon storage dominating the ecosystem service values and swamping opportunity costs. Other benefits associated with conservation were more modest and exceeded costs only in protected areas and indigenous reserves. We used this cost-benefit information to show that one potential corridor between two large forest patches had net benefits that were three times greater than two otherwise similar alternatives. Spatial cost-benefit analysis can powerfully inform conservation planning, even though the availability of relevant data may be limited, as was the case in our study area. It can help us understand the synergies between biodiversity conservation and economic development when the two are indeed aligned and to clearly understand the trade-offs when they are not.

## Abstract 2

A spatially explicit assessment of the economic costs and benefits of conservation in Paraguay highlights the challenges and potential utility of such an approach for understanding the value of biodiversity.

## Introduction

Investments in biodiversity conservation must be strategically allocated, because resources are severely limited [1]. As a result, approaches for designing conservation plans that systematically represent a region's biodiversity have proliferated and become ever more sophisticated [2,3]. Although the biological aspects of these approaches have advanced rapidly, relatively little attention has been paid to the economic side of conservation planning (i.e., the science of systematically prioritizing conservation interventions), even though planning invariably involves both costs and benefits. Understanding costs— including land prices, management costs, and opportunity costs (i.e., foregone alternatives)—will help us to allocate scarce dollars most efficiently [4]. And understanding benefits—“ecosystem services” such as flood control from wetlands and carbon sequestration from forests—will help us to estimate the economic value of lands identified for conservation and to identify who may be willing to pay for these services [5].

Cost-benefit analyses, where the economic costs and benefits of a proposed policy or project are tallied and used to inform decision making, are widely used in a variety of issue areas, including the health, safety, transport, and development sectors [6]. These analyses can indicate whether the aggregate benefits of a policy decision outweigh the aggregate costs, and they can help quantify the resulting economic gains and losses among groups. Such information can be crucial in making efficient decisions about how to best allocate scarce resources in pursuit of various policy objectives [6].

Conservation biologists have been slow to incorporate these cost-benefit approaches into their work [7,8], but some recent studies demonstrate the potential power of economics to inform conservation decisions. On the costs side, economists have shown that conservation plans that incorporate costs can represent equal or greater levels of biodiversity with dramatically fewer resources than plans that do not consider costs [9–12]. Global-scale analyses have illustrated that the costs needed to establish and manage protected areas vary enormously among countries [13]. Recent calls for more work on the costs of conservation indicate that these findings are slowly penetrating the planning literature [4,14]. On the benefits side, there has been an increased awareness of the economic value of ecosystem services provided by natural systems [5,15]. Quantifying these values, however, remains complex and has become a major area of research in both environmental and ecological economics [16,17]. New techniques have led to a much greater ability to quantify economic values associated with natural ecosystems in a wide variety of contexts [18,19]. Paralleling this research on valuation has been an increased interest in developing mechanisms that compensate landowners for the ecosystem services their lands provide [20,21].

Despite these advances, explicit analyses of economic costs and benefits have yet to become widely incorporated into conservation planning exercises. In part, this is because conservation planning is inherently spatial and thus presents special challenges for the quantification of both costs and benefits. For costs, spatially explicit data on land prices at the necessary resolution are lacking for many parts of the world, in which case they must be modeled [22,23]. For benefits, the biophysical delivery of ecosystem goods and services must first be spatially quantified, a difficult task in itself [24,25], and then these ecosystem services must be assigned an economic value in a spatially explicit manner. This requires knowledge of who the beneficiaries are, where they reside, how they perceive the value provided by an individual ecosystem service, and how the spatial pattern and scale of an ecosystem service affects the resulting economic values at the scale of interest [26].

In spite of the numerous challenges, cost-benefit analysis could provide novel insights into conservation planning. First, it would allow the spatial distribution of benefits and costs to be compared to the distribution of biodiversity, allowing us to locate areas of value for both biodiversity and people (“win-win” areas for conservation), and also allowing us to identify areas of conflict or tradeoff, where net economic benefits of ecosystem conservation are low but biodiversity values are high (and vice versa). Second, a spatial cost-benefit analysis would highlight which areas have the greatest benefits per unit cost, thus allowing the most efficient targeting of efforts towards conservation. Third, maps of ecosystem services would help identify suppliers and consumers of ecosystem services, allowing the identification of efficient and equitable payment mechanisms to fund conservation projects [21]. Finally, an improved understanding of the spatial distribution of the benefits of ecosystem conservation, relative to costs, would indicate in which areas conservation makes economic sense, providing an economic case for conservation to bolster moral and aesthetic arguments [27,28]

Here we report on a cost-benefit analysis that incorporates spatially-explicit valuations of ecosystem goods and services along with opportunity costs of conservation. We selected five ecosystem services and quantified their economic values across a landscape in eastern Paraguay. The beneficiaries of these ecosystem services range from local individuals to citizens of countries far away from the study site, and therefore our perspective is social; we are estimating the benefits of ecosystem services to society as a whole, although we are careful to maintain separate estimates for each class of beneficiaries in discussions and policy implications. We compared the ecosystem service values to the cost of conserving the natural habitat that underlies their provision [29] and asked which areas would pass a cost-benefit test. We also compared three corridor options within the landscape and asked how a consideration of both the costs and benefits can inform decisions on which to pursue as a conservation objective.

Because we are unaware of previous studies that have estimated the economic costs and benefits of conservation in a spatially explicit manner, we emphasize the preliminary nature of this study and highlight the associated assumptions, pitfalls, and challenges. In particular, we note that this study is based on a utilitarian view of conservation, where benefits and costs are assessed in purely economic terms. We do not consider here deeper issues of “value,” such as the intrinsic value of nature and ethical issues associated with conservation. These values, while impossible to quantify in economic terms, are clearly fundamental to conservation of the natural world. The analyses presented here are meant to complement, not replace, more profound considerations of the value of biodiversity.

## Materials and Methods

### Study Site: Mbaracayu Biosphere Reserve, Eastern Paraguay

The Mbaracayu Forest Biosphere Reserve in eastern Paraguay is within the highly threatened Upper Parana Atlantic Forest ecoregion (Figure 1). In 1973, this reserve (which follows the boundaries of the Upper Jejui watershed, an area of 2,920 km^2^) was 90% forested. In 2004, the area of forest in the watershed had been reduced to 56% and, with the exception of a central core protected area and several forests on adjacent private lands, was becoming highly fragmented. As of 2004, there were three dominant anthropogenic land uses in the region: smallholder agriculture (12% of land surface), large-scale cattle ranching (14%), and soybean production (2.4%). Residents of the region include the Ache, an indigenous group who have lived in the forests of the region for at least the past several hundred years and possibly much longer [30]. Although all Ache groups have been settled onto reserves, many still continue to use the forest for traditional hunting and gathering purposes, including within the core protected area, where this is legally permitted. Indigenous Guarani tribes also live in several settlements in the region. Campesinos (smallholder farmers) live in government sponsored *colonias* and practice small-scale farming of both cash and subsistence crops. Large landowners, including many Brazilian nationals, own huge tracts of land (thousands of hectares) for both cattle ranching and soybean production.

**Figure 1:**
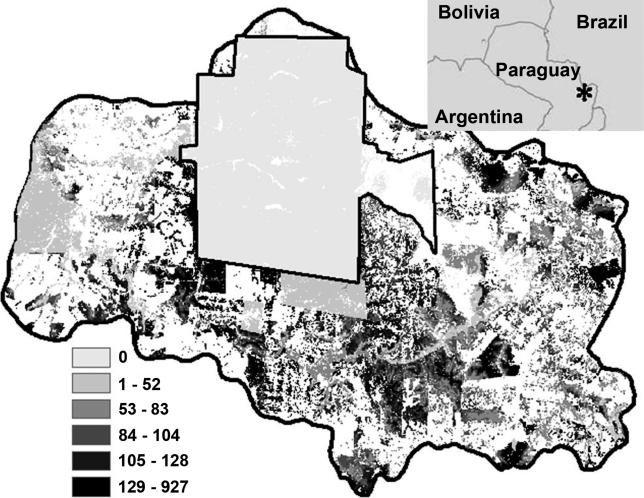
The Mbaracayu Biosphere Reserve and its Location in Eastern Paraguay The outer line delineates the border of the Reserve, and the roughly rectangular core protected area is outlined within it. Areas in white are nonforest areas (mostly agricultural) and were not considered. Areas in shades of gray to black are forested; darker shades represent areas with higher opportunity costs, which are net present values in US$ per hectare (adapted from [29]).

### Estimating Costs

In a previous study, the opportunity costs of conservation associated with the dominant anthropogenic land uses were modeled for the Mbaracayu Forest Biosphere Reserve [29]. Opportunity costs of conservation were defined as the expected agricultural value of each forested parcel of land, because this was an estimate of the best alternative economic use of the land. Opportunity costs were calculated as the probability that a given parcel would be converted from forest to an agricultural land use, multiplied by the expected net benefits from that land-use type, and then summed over all land uses. Conversion probabilities were estimated based on past patterns of forest conversion to known agricultural land uses, and the net economic benefits of the various types of land uses were derived from regional estimates. This resulted in spatially explicit estimates of opportunity costs of conservation for each hectare of forest in the Biosphere Reserve.

To check the accuracy of these estimates, per-hectare opportunity costs were compared to actual property values for a sample of 20 properties within the Biosphere Reserve. A strong correlation between predicted land values and actual property prices was demonstrated [29]. In particular, predictions using discount rates of between 15 and 25% were unbiased estimators of actual property values. We therefore used this cost layer as a baseline against which to compare the value of ecosystem services, with a discount rate of 20% applied uniformly throughout the landscape.

Because the opportunity cost layer was developed in part using information on past conversion rates, the core protected area of the Biosphere Reserve has zero opportunity costs of conservation, because there has been no detectable deforestation in the area over the past 15 years. This illustrates a weakness of the methodology: it is based on deforestation rates and patterns observed in the past. If the conditions under which this pattern occurred were to change (e.g., a change in zoning that removes the core area's protected status), the estimated opportunity costs layer would not reflect this change and would need to be recalculated. We did not consider other potentially important measures of conservation cost (e.g., management costs), because we had no way to estimate these across the landscape.

### Estimating Benefits

The basis for our assessment of ecosystem service values was a forest cover map of the biosphere reserve. We used Landsat imagery and ground data from a separate project [31] to map forest cover types (methods are described in [23]). The final map recognizes six forest types, which were derived from agglomerations of Ache vegetation classifications: high forest, low forest, big bamboo forest, bamboo understory forest, vine undergrowth forest, and swamp [23].

Using this forest map, we assessed five different ecosystem services: sustainable consumption of bushmeat, sustainable timber harvest, bio-prospecting (value for new pharmaceutical products), existence value, and carbon storage. For each of these services, we describe below the beneficiaries, the methods used to map out associated economic values, and the assumptions we made. For some services, site-specific valuation information did not exist; in these instances, we took a benefits transfer approach to estimating values. A discussion of benefits transfer (i.e., the transfer of economic value estimates derived at one geographical location to a different location) is outside the scope of this paper; however, we note that the development and reliability of such methods is greatly debated in the environmental economics literature [32–34].

### Bushmeat

Hunting for wild meat is the most important economic activity of the Ache [35] and is also practiced by campesinos and large landowners. To map out the economic value of bushmeat, we integrated biological information on important game species [31], forest type classes of the Biosphere Reserve, and prices of domesticated meat in the region's primary market town (Villa Ygatimi). Habitat associations of game species were modeled using logistic regression equations modified from [31]. Maps of predicted probability of occurrence were constrained by minimum forest areas required for estimated home ranges of the various species, such that probability of occurrence was set to zero for forest fragments below a species-specific minimum threshold. For each species, we multiplied this constrained expected probability of occurrence by the average body size for an individual, then by the size of the group that individuals of a species are typically found in, and then by the species-specific fraction of biomass that can be sustainably harvested (Table 1). Summed over all species, this resulted in the expected number of kilograms of bushmeat a forested cell could provide. This analysis is highly simplified and does not include complexities such as species population dynamics, hunter behavior, and household demand that more sophisticated studies account for [36].

**Table 1:**
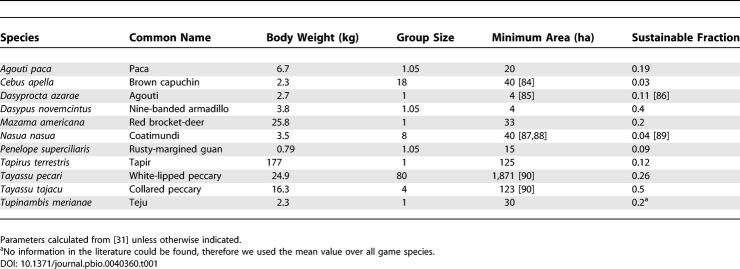
Game Species Considered in the Analysis of Bushmeat Economic Values in the Mbaracayu Forest Biosphere Reserve, Paraguay

Because bushmeat is not traded by either the Ache or by campesinos, we assumed that in the absence of meat from wild animals, individuals would have to substitute domestic meat to meet their protein requirements. We therefore multiplied the local market price of a kilogram of beef (US$1.44 as of May 2005) by the expected kilograms of bushmeat that a forested cell provides, and considered this the economic value of the potential sustainable flow of bushmeat that is expected from each hectare of forest in the region.

We assume here that hunters actually do harvest bushmeat at the levels and spatial patterns that our analysis maps out. However, we have not compared this predicted pattern of offtake from what actually occurs. Hunting by the Ache in the Mbaracayu Forest Reserve is substantially lower than the theoretical maximum sustainable offtake, but speculation is that poaching by campesinos is nonetheless resulting in the depletion of certain species [31]. For the rest of the forests in the Biosphere Reserve, we have no data on hunting rates, but anecdotal evidence suggests that the larger game species such as tapirs and peccaries are much less common than in the core protected area, likely due to a combination of forest fragmentation and heavy hunting pressure. Therefore, our estimates of the economic value of bushmeat are likely overestimates for at least some areas and should be cautiously regarded as the value of a potential flow of bushmeat from the region's forests under a sustainable management system.

### Timber Harvest

Local residents, especially large landowners with access to machinery and labor, harvest a variety of high-value timber species in forests of the Biosphere Reserve (Villa Ygatimi, the biggest town in the watershed, has several sawmills in operation). What we assessed is the potential sustainable flow of a limited timber harvest from forests of the region, similar to the analysis of bushmeat. We assume that such a harvest would not change the structure of the forest such that the provision of other ecosystem services we considered would be affected.

To estimate the economic value from sustainable timber harvests, we used data from research that calculated the value of standing merchantable timber from various species in the Biosphere Reserve [37]. This is the value to the landowner of a standing tree of an economically valuable species and does not include the value added through harvesting and sales to the mill. Using information on 16 tree species, we calculated an average per-tree value of US$6.87. We were unaware of any studies that had assessed the sustainability of reduced-impact logging schemes in the Mbaracayu area, therefore we used guidelines from lowland Bolivian forests and assumed a sustainable harvest rate of four trees per hectare with a 30-year harvest cycle [38]. We assigned a timber value of zero to the core protected area, because harvesting is not permitted there. Elsewhere, we assumed all forest types were equally valuable for logging, with the exception of big bamboo and low forests types, which we assumed did not contain any valuable timber species.

### Bioprospecting

The value of natural habitats (especially tropical forests) as providers of potential new medicines that may benefit humanity has long been proposed as a compelling reason for conservation [39]. A number of studies have attempted to quantify the value of tropical forests as potential storehouses of undiscovered pharmaceutical products or precursors [40–42] and some of these have suggested very high potential values [43].

We used data from a study that assessed the willingness to pay (WTP) of pharmaceutical companies for the potential of tropical forests to contain precursors to new marketable drugs [40]. These researchers estimated the value of the marginal species; i.e., the value that each additional species contributed to the willingness-to-pay of companies, restricting potential new discoveries to plant species and making assumptions regarding the costs of screening and research and development. We followed their procedures but recalculated the marginal value of a species using a discount rate of 20% (to conform with the discount rate used in the rest of our calculations), and we then used this to calculate the value of a marginal hectare of habitat, again following the methods of [40]. We assumed that all forests in the Biosphere Reserve have equivalent per-hectare species endemism levels, whereas it is highly likely that forests in the core protected area are much more diverse than the unprotected and often degraded forests found throughout the rest of the region [44].

### Existence Value

Not only does the natural world provide tangible goods and services such as food, fiber, and nutrient cycling, but it also serves as a source of wonder and inspiration for many people [5,15]. Economists call this existence value, and studies using sophisticated survey methods have attempted to quantify the WTP of various groups of people to conserve natural habitats, even if these provide them with no direct use value [45–47]. To estimate this value for forests in our study area, we used data from a synthesis of global economic values of forest ecosystems [48]. This synthesis indicates considerable variability in estimates of existence value for various forest regions, which is associated with differences in both methodology and attributes of the forests that were valued. We chose to be conservative and therefore selected a value of US$5/ha per annum for household WTP for the existence of forests in the Mbaracayu region, a value based on debt-for-nature swaps for all tropical forests. We assume that the forests in our region are representative of tropical forests in general, and that they would qualify for additionality based on rapid conversion rates outside protected areas. We discounted these values into the future and applied them to all forests outside the core area, because this area is already protected as a nature reserve.

### Carbon Storage

Forests contain large quantities of carbon that may be released into the atmosphere if they are cleared, resulting in increased carbon dioxide (CO_2_) emissions. In theory, therefore, standing forests are economically valuable if they are at risk of conversion, because preventing conversion also prevents potentially substantial rises in CO_2_ emissions. We considered the economic value of forests for the avoided emissions of carbon that is currently stored in aboveground biomass. We used information from replicate 0.04-ha plots to calculate average height, diameter at breast height (DBH), and stem density of trees within each forest type (Table 2), and we then estimated the carbon content by calculating total tree biomass from allometric equations [49] and multiplying by 0.5 (the fraction of carbon in biomass; [50]). At the time of writing, carbon was trading in various markets at prices ranging from US$1.80 to US$25.50 per tonne CO_2_ (http://www.ecosystemmarketplace.com; accessed January 3, 2006). We used a conservative estimate of US$2.50 per tonne CO_2_ (this translates to US$9.17 per tonne of carbon) to calculate the economic value of carbon contained in the standing biomass of the various forest types of the region. This value of US$9.17 per tonne carbon is also around the midpoint of the range of estimates for the social damage value of carbon emissions [51–53].

**Table 2:**
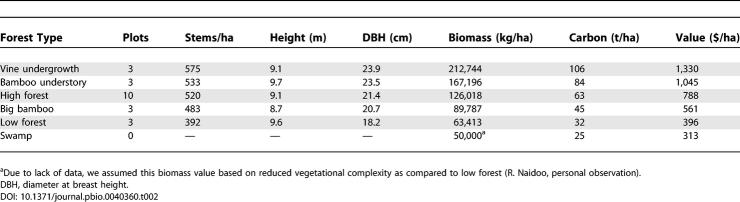
Information Used to Calculate the Economic Value of Carbon Storage for the Various Forest Types in the Mbaracayu Forest Biosphere Reserve, Paraguay

A number of assumptions regarding the science and policy context of carbon emissions are needed for these economic values to be viable. The science of CO_2_ emissions and land-use change is evolving; we avoided dealing with dynamic and/or contentious issues such as the permanence of carbon in forests [54] and potential changes in carbon sink functioning as climate changes [55]. On the policy context front, we made three significant assumptions. First, we assumed that avoided deforestation, which is not currently part of the Clean Development Mechanism (CDM) of the Kyoto Protocol, is a valid means of reducing CO_2_ emissions. At least some groups are pushing strongly to include avoided deforestation in any deal that extends past the current Kyoto Protocol lifespan; avoided deforestation would then join reforestation and afforestation as legitimate means to reduce CO_2_ emissions [56]. Second, we assumed as a baseline that all forests outside the core protected area were facing imminent deforestation without an intervention project that invests in the carbon content of these forests. This assumption is necessary to satisfy the “additionality” criterion [57] of the Kyoto Protocol, and given the high rates of deforestation and continuing profitability of soybean farming in the area, is realistic. Third, we assumed the existence of a willing buyer who would invest in the area's forests for their carbon value, and we did not consider the often high transaction costs that can reduce the viability of small CDM projects [58]. Lastly, we took a “social damage” accounting standpoint that reflects the damage avoided by society at large from increased CO_2_ emissions; we therefore assumed a one-time initial payment for avoided emissions that results in permanent forest protection. More sophisticated accounting systems that are tightly linked with CDM rules and regulations [59,60] are not considered here.

### General Assumptions

In addition to assumptions specific to each ecosystem service, we made a number of additional simplifications for the analysis to be tractable. First we assumed a universal discount rate of 20% for all service valuations, so as to be consistent with the discount rate that was used to calculate the opportunity costs of conservation (see Discussion for considerations of the implications of this assumption). Second, when taking a benefits-transfer approach, we used a combination of marginal and average values for our calculations of ecosystem service benefits; marginal values are the more appropriate metric but are usually more difficult to determine unless site-specific valuation studies exist. Third, we assumed that the provision of ecosystem services was independent within a parcel of forest; e.g., that the sustainable harvest of bushmeat or timber does not affect values associated with bioprospecting or carbon storage. The degree to which interactions affect ecosystem service provisioning and value is an empirical question that we did not have the data to address. Finally, we were unable to model spatial interdependencies of ecosystem services among parcels of land. True spatial modeling (i.e., that explicitly considers the effect of one parcel or patch on another; see [61] for spatial modeling of resource exploitation) for ecosystem service valuation was beyond this analysis because of limitations in our theoretical understanding of spatial interdependencies and a lack of relevant empirical data. Addressing these deficiencies in the empirical work is an important avenue for future research.

## Results

### Opportunity Costs

Opportunity costs of conservation in the Mbaracayu Forest Biosphere Reserve were heterogeneous and varied across almost three orders of magnitude, from US$0 to US$927/ha [29] (Figure 1). Variation was related to a number of factors, such as land tenure (protected areas and indigenous reserves had low opportunity costs due to low conversion rates), slope (steeper slopes had lower deforestation rates and therefore lower costs), and soil type. Most of the high value land was concentrated in the eastern part of the Biosphere Reserve, because of its high potential for soybean farming, which was the most profitable land use in the region. When aggregated to the level of individual properties, modeled costs were strongly correlated with actual property values, even after correcting for the effects of property size and forest cover. These results are discussed in more detail in [29]. Over all forests (including the core protected area where values are essentially zero), the average opportunity costs of conservation were around US$60/ha.

### Ecosystem Services

As with costs, the summed per-hectare values of the five ecosystem services ranged over three orders of magnitude, from US$2/ha up to US$1,045/ha (Figure 2A). Bushmeat varied from US$0/ha to US$18.50/ha (Figure 2B); smaller blocks of forest were assumed not to contain larger game species and therefore patch size was the strongest correlate of per-ha value. For sustainable timber harvest, bioprospecting, and existence value (Figure 2C–2E), we were able to apply only a single value to all qualifying forest areas, either because data on spatial variability were lacking (timber harvest, bioprospecting), or because values were spatially homogeneous at the scale of this landscape (existence value). Carbon storage varied among forest types (Figure 2F), with vine undergrowth and bamboo understory forests having the highest carbon storage values and low forest and swamp having the lowest values.

**Figure 2:**
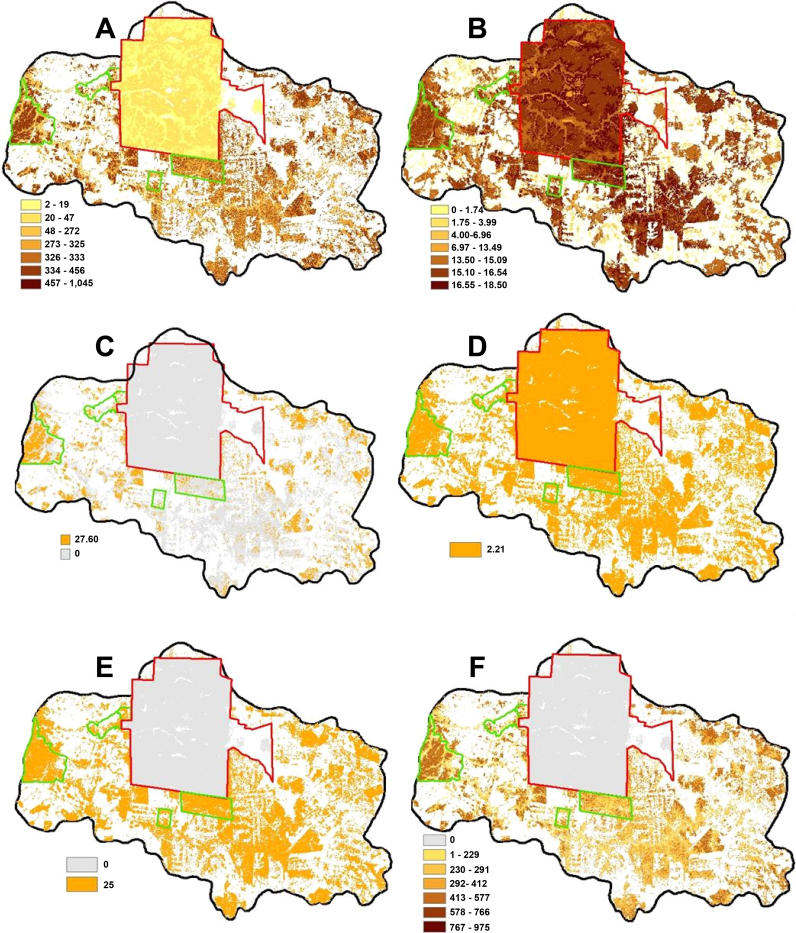
Economic Values (Net Present Values in US$ per Hectare) Associated with Forest Ecosystem Services across the Mbaracyau Forest Biosphere Reserve (A) Sum of all 5 services. (B) Sustainable bushmeat harvest. (C) Sustainable timber harvest. (D) Bioprospecting. (E) Existence value. (F) Carbon storage.

On an average per-hectare basis, carbon storage was by far the most highly valued ecosystem service, with a value of US$378/ha. The next most valuable service was sustainable timber harvest (US$27.60/ha), followed by existence value (US$25/ha), bushmeat harvest (US$15.59/ha), and bioprospecting (US$2.21/ha). Economic values in the core protected area were much lower than in other forests, in part because its protected status disqualified it from accruing existence and carbon values, and in part because the activities permitted within (sustainable bushmeat harvest and, hypothetically, bioprospecting) were very low value.

### Conservation Planning

Whether a particular forested area passed a spatial cost-benefit test for conservation was dependent on how many of the five ecosystem services were included (Figure 3). When considering only ecosystem services that were the most local or private in nature (i.e., bushmeat, timber, and bioprospecting for pharmaceutical products), only forests in the core protected area and indigenous reserves had benefits that exceeded opportunity costs (Figure 3A–3C). After existence value was added, 19% of forests outside of the core protected area would pass a cost-benefit test, but most of these still lie in indigenous reserves (Figure 3D). Finally, when carbon values were added to the local services, ecosystem service values of virtually all forests (98%) exceeded the opportunity costs of conserving them (Figure 3E).

**Figure 3:**
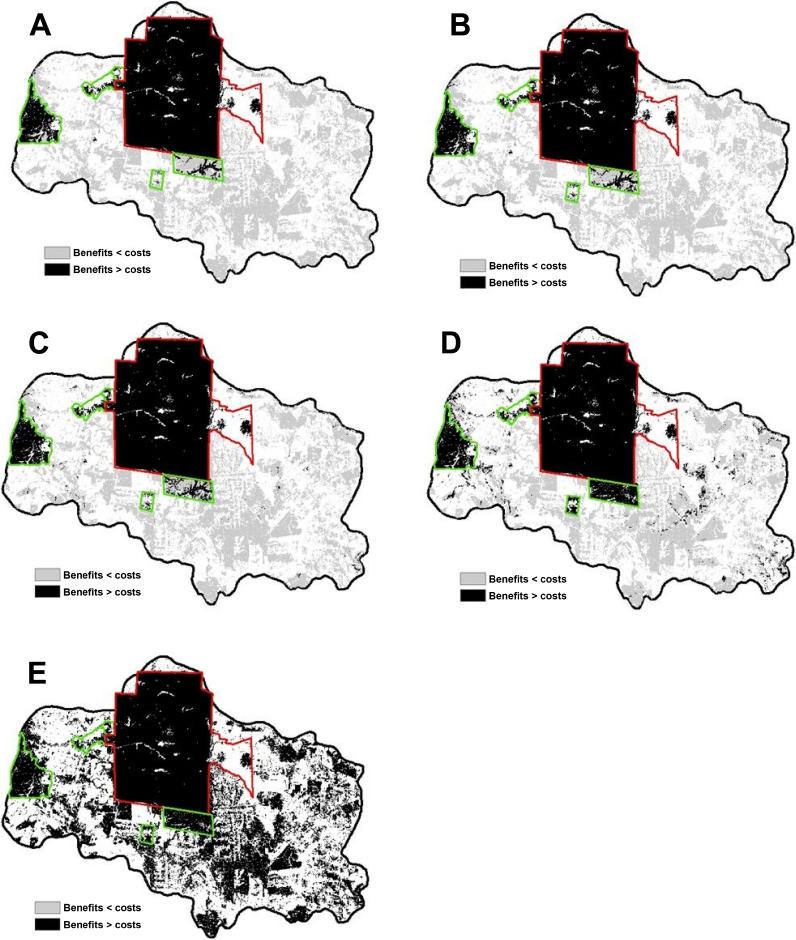
Location of Forested Areas in the Mbaracayu Biosphere Reserve where Economic Benefits Exceed Opportunity Costs (Shown in Black) Each panel represents calculations considering a different set of benefits. (A) Sustainable bushmeat harvest; (B) bushmeat + sustainable timber harvest; (C) bushmeat + timber + bioprospecting; (D) bushmeat + timber + bioprospecting + existence value; (E) bushmeat + timber + bioprospecting + existence value + carbon storage.

Mapping the ratio of costs and benefits across the landscape makes clear the degree to which one exceeds the other for each parcel of land (Figure 4). Using this cost-benefit information, we considered three potential corridors for improving connectivity to the core protected area in the landscape [62] (Figure 4). All corridors had similar total areas and forested areas (Table 3). We summed the economic values associated with costs and benefits for all cells in each corridor and calculated the associated net benefits. When all five ecosystem services were considered, benefits of all three corridors greatly exceeded costs (Table 3). When only services accruing locally were considered, however, benefits were less than opportunity costs for all corridors. Of most interest, the difference between local benefits and costs varied greatly; corridor 3, the largest in total area and second largest in forest area, had much lower costs than the other two corridors and had higher local benefits. Therefore the costs not offset by the value of local ecosystem services were almost three times lower for corridor 3 as compared to the other two corridors (Table 3).

**Figure 4:**
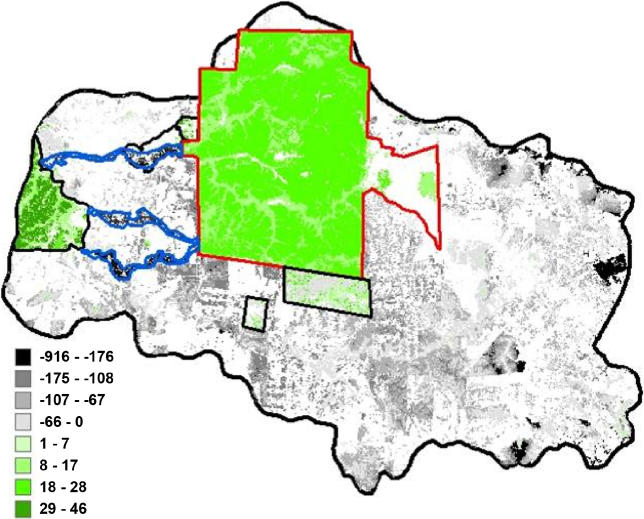
Differences between Economic Costs and Benefits of Conservation across the Mbaracyau Forest Biosphere Reserve Green represents areas where economic benefits exceed costs (both as net present values in US$ per hectare), based on bushmeat + timber + bioprospecting values. Gray/black areas are where costs exceed benefits. Darker shading indicates greater deviation from zero in either direction. Three potential corridors connecting the core protected area and the large indigenous reserve to the west are outlined in blue.

**Table 3:**
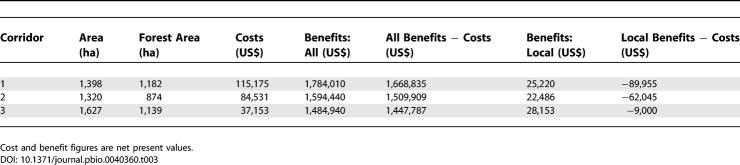
Comparison of the Area, Costs, and Benefits of Three Hypothetical Corridors Connecting the Core Protected Area of the Mbaracayu Forest Biosphere Reserve with the Large Indigenous Reserve to the West

All of these results depend on the accuracy of the parameter estimates for each service. The sensitivity of the cost-benefit results to parameter uncertainty appears relatively low, however (Table 4). When considering only local ecosystem services, a 20% reduction in key parameter values for each of the three local ecosystem services resulted in only about a 5% decrease in cells passing a cost-benefit test. When considering all services, sensitivities were even lower, and only large drops in the price of carbon would result in significant differences in the results of the spatial cost-benefit analysis (Table 4).

**Table 4:**
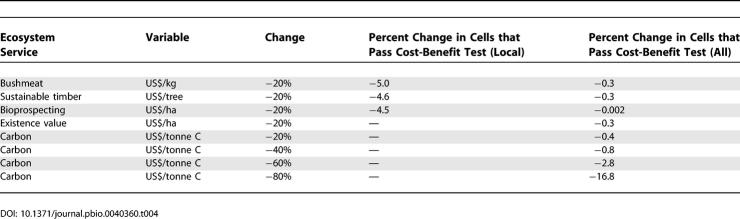
Sensitivity of Cost-Benefit Results to Changes in Key Parameters of Ecosystem Service Value Calculations

## Discussion

We found that both benefits and costs of conservation varied enormously across the Mbaracayu Biosphere Reserve. This spatial information can inform conservation and land use decisions but is currently lacking in most conservation planning exercises. We found that economic benefits of conservation are substantial and, depending on which services are counted, outweigh costs in certain areas. In these areas, financial mechanisms that capture the economic value of ecosystem benefits can help finance conservation, freeing up resources for investment elsewhere. We also found that accounting for the costs and benefits of conservation can help illuminate economic trade-offs for specific decisions such as the placement of corridors. These results argue for increasing research into spatial cost-benefit analyses for conservation, so that economic information can complement the biodiversity layers typically used in conservation planning.

The ratio of benefits to costs varied greatly across the Mbaracayu region (Figure 4). In the core protected area and indigenous reserves, opportunities for agriculture and other land uses are severely restricted, so opportunity costs as we defined them were quite low [29]. As a result, benefits exceeded costs in these places, even when only the lowest valued services were considered. In general, however, the economic benefits of ecosystem services that accrue locally (bushmeat, timber harvest) were small and offset only a fraction of the costs of conservation (Figure 3A–3C). This accords with recent findings that suggest that nontimber forest products have very low economic values that cannot compete with alternative land uses that involve conversion of natural habitat [63,64].

In contrast, the value of forests for carbon storage, a global value reflected on internationally-traded markets, dominated the aggregate economic value of ecosystem services and exceeded everywhere the associated opportunity costs of conservation. Other studies have pointed out that internalizing carbon values through the development of appropriate financial mechanisms can ultimately determine whether or not conservation is profitable in tropical forests [65,66]. This was the case in our study, even though we used a price (US$2.50 per tonne CO_2_) that was at the low end of the values that are trading on various markets (i.e., US$1.80 to US$25.50 per tonne CO_2_). Using a higher price such as the one that is traded on the European market for carbon (US$25.50; http://www.ecosystemmarketplace.com; accessed January 3, 2006) would only have further increased the value of carbon relative to other services and opportunity costs of conservation. Alternative definitions of which areas pass the additionality criterion (i.e., the requirement that forests that are conserved through a carbon investment would otherwise have been cut; see Materials and Methods) would not change our qualitative result that payments for carbon storage services could preserve a substantial amount of the region's forests.

The three hypothetical corridors we considered in the Mbaracayu landscape were similar in size and would achieve the same goal of connecting two existing large forest reserves. However, the shortfall in local ecosystem service benefits, relative to costs, varied greatly and was much lower for corridor 3, which suggests that investing in corridor 3 would be the most efficient use of scarce conservation dollars, as long as biodiversity targets and others features of interest are similar among corridors. This result illustrates the inefficiency risks of using area as a proxy for cost in conservation planning [11]. Actual economic cost data, instead of area or other proxies like human population density [67] or aggregate measures of human conflict [68], can more directly inform land-use decisions, which almost always include financial considerations. Coupling spatial estimates of cost with those of economic benefits of ecosystem services will further inform decisions by indicating what proportion of expected costs might be offset by payments for environmental services [21]. Although local benefits did not outweigh costs for any of the three corridors (Figure 4 and Table 3), the quantification of benefits and costs is nevertheless useful because it indicates the financial shortfall that environmental service payments would not offset.

In our analyses we were only able to consider five ecosystem services provided by standing forests. We were unable to quantify values associated with watershed services [69], recreation [70], option values [71], or quasi-option values [72]. Our valuation estimates for ecosystem services are therefore only a lower bound and might be significantly higher after a more complete treatment of the services provided by forests in this area.

To be consistent in our analyses, we chose to use a constant discount rate of 20% in calculations for the opportunity costs and for each of the individual benefits (ecosystem services) associated with forest conservation. This rate was adopted from [29], where it was shown that modeled values of opportunity costs were consistent with actual property values at 20%. While 20% is a reasonable figure for use in a developing country study site such as Mbaracayu, it is likely to reflect a much higher rate of time preference than those of developed-country beneficiaries of services such as existence value, carbon storage, and bioprospecting. If this is the case, we have probably underestimated the economic values of the latter services by using too high a discount rate. However, lowering the discount rate for these services would not lead to qualitative changes in our results. Existence value and especially carbon storage were the most valuable ecosystem services, and together, their values exceeded opportunity costs in all forested areas. Lowering the discount rate for these services would only emphasize this trend even farther. In the case of bioprospecting, dollar values were so low that changes in the discount rate would not significantly change cost-benefit results. Nevertheless, specifying the appropriate discount rate in cost-benefit analyses is a key challenge and should be carefully considered when making cost-benefit calculations [73].

For each of the services we investigated, we were forced to make simplifying assumptions due to lack of data on biophysical distribution, economic valuation, or both. For bushmeat, we had detailed information on game species distributions, but information on hunting patterns throughout the region was lacking, and we assumed a sustainable offtake level, which may not be warranted [31]. For carbon storage, we ignored belowground biomass and assigned no storage value to agricultural systems, even though some carbon is stored in these areas of reduced vegetational complexity [74]. For timber harvest, we assumed certain forest types to be completely void of merchantable tree species, whereas for bioprospecting and existence values, we assumed that forest values were well represented by very coarse regional estimates. For some services the beneficiary population and its spatial distribution was highly uncertain, and in each case we had very few (if any) valuation studies from which to draw on in terms of assigning prices to services. This highlights a serious gap in our knowledge of environmental valuation and suggests that much original research will need to be done to value ecosystem goods and services in novel contexts.

Sensitivities of cost-benefit results to changes in key parameters in the valuation of each ecosystem service appeared low (Table 4). This was because independent 20% reductions in any one ecosystem service parameter were not great enough to change the aggregate ecosystem service value that is derived primarily from the sum of the other, nonchanging services. This was especially true when we considered all five ecosystem services, instead of only those accruing locally. Although we did not conduct a comprehensive sensitivity analysis across all valuation parameters simultaneously, our results are likely to be robust within estimation errors of 20% of parameter values.

Our analysis emphasizes several issues that may arise when evaluating the economic value of ecosystem services in a spatially explicit manner. First, identifying the beneficiaries for each ecosystem service is difficult because of the wide range of spatial scales at which they may be distributed. For example, some ecosystem services only benefit nearby users (e.g., pollination; [75]) or those who are directly downstream (e.g., water provision; [76]), whereas others benefit a much larger group of people at regional or even international scales (e.g., carbon storage; [77]). Second, the same service may provide different values to different groups of people [65]. Values can vary due to spatial proximity [78], socio-economic status [79], or cultural factors [80]; this variation may limit the possibility of benefits transfer and therefore field studies to understand site-specific ecosystem service values are critical. Finally, our spatial models were stronger on the biophysical side than on the economics side: we were unable to incorporate models of human behavior that could drive changes in prices, and therefore value, through time or space. This made it difficult to calculate marginal values, because we were unable to trace out spatial supply and demand curves, and so we were left with averages based on total values, which may be misleading when applied to land-use decisions on the margin [27]. More research in this aspect of ecosystem service valuation is sorely needed.

Although mapping and valuing ecosystem services can help to inform planning efforts, it is not sufficient to motivate conservation. For most ecosystem services, financial mechanisms and institutions (e.g., markets, subsidies) do not exist to capture values and compensate landowners for bearing the costs of providing them [21]. An increasing number of examples demonstrate the potential of such mechanisms, including payments for services from forests in Costa Rica [81], water purification in New York [82], and water table maintenance in Australia [83]. For all but these and a few other exceptions, however, payment schemes for services outside traditional markets are typically absent. Without such mechanisms, many economic values associated with natural habitats will remain outside the calculus of agents who actually make land-use decisions.

The preceding is especially true in the case of carbon storage values and illustrates the gap that needs to be overcome to provide real economic incentives to people living in or near threatened forests such as those at Mbaracayu. If avoided deforestation were to be included in the CDM of the Kyoto Protocol [56] and if the forests in this area qualified for a carbon intervention project, the economic value of standing forests would greatly exceed the value associated with clearing and farming them. This result would also be true (at least for most of these forests) if a mechanism were in place to capture the high existence values that residents of developed countries place on forests of the developing world. In each case, it is clear that although local economic values of forests cannot compete with the profits to be made by clearing them, international transfer mechanisms that translate developed country values into dollars for developing world economic actors could have an enormous impact on conservation. As seen by difficulties in ratifying the Kyoto Protocol, the challenges in creating and implementing such institutions are formidable. Yet doing so would ultimately be of tremendous benefit to conservation.

Despite the issues and difficulties we have discussed, spatially-explicit information on economic benefits and costs of conservation appears highly useful to conservationists and policy makers. Ecosystem services often hold significant economic value, but they remain undervalued within policy decisions because they are poorly understood and typically external to markets. As a result, cost-benefit analyses are biased toward development over conservation, and planning efforts miss potential “win-win” areas and associated opportunities to finance conservation in innovative ways. Maps such as those we have developed here can demonstrate the economic value of conserved lands, locate these win-win areas, and motivate payments for environmental services from consumers to suppliers.

The economic considerations we describe here in no way compete with or override moral and aesthetic arguments for conservation. Maintaining the diversity of life on Earth is essential in its own right, and conservation efforts must first and foremost target biodiversity, not a favorable benefit:cost ratio. Deepening our understanding of the economic aspects of conservation, however, will allow decision makers to realize the synergies between biodiversity conservation and economic development when the two are indeed aligned and to clearly understand the trade-offs when they are not.

## Abbreviations

CDM: Clean Development Mechanism
WTP: willingness to pay
